# Biomolecular Pathways Linking Preoperative Chronic Stress to Postoperative Cardiovascular Dysfunction in Noncardiac Surgery

**DOI:** 10.3390/biom16081183

**Published:** 2026-08-13

**Authors:** Predrag Jancic, Ivana Kovac, Halil Emre Demirtas, Nebojsa Nick Knezevic, Graham Trevor Lubinsky

**Affiliations:** 1 The Wright Center for Graduate Medical Education, Scranton, PA 18505, USA; jancicp@thewrightcenter.org (P.J.); demirtash@thewrightcenter.org (H.E.D.); 2 Department of Anesthesiology, Advocate Illinois Masonic Medical Center, Chicago, IL 60657, USA; kovac.ivana29@gmail.com; 3 Department of Anesthesiology, University of Illinois, Chicago, IL 60612, USA; 4 Department of Surgery, University of Illinois, Chicago, IL 60612, USA; 5 Department of Anesthesiology, School of Medicine, Wake Forest University, Winston-Salem, NC 27157, USA

**Keywords:** chronic stress, allostatic load, glucocorticoid resistance, hypothalamic–pituitary–adrenal axis, myocardial injury after noncardiac surgery (MINS), perioperative risk assessment, psychological distress

## Abstract

Postoperative cardiovascular complications remain a leading cause of morbidity and mortality after noncardiac surgery, yet current risk models do not incorporate psychosocial stress. With over 300 million noncardiac surgeries performed annually worldwide and a substantial burden of perioperative cardiovascular complications, preoperative chronic stress is increasingly recognized as a potentially modifiable risk factor. Chronic stress produces HPA axis dysregulation, glucocorticoid resistance, sympathetic activation, inflammation, endothelial dysfunction, and hypercoagulability. These pathways overlap with the mechanisms underlying perioperative myocardial injury, arrhythmogenesis, and venous thromboembolism. Prospective data demonstrated that preoperative psychological distress independently predicted 30-day cardiovascular complications and 1-year mortality after noncardiac surgery. Allostatic load studies in noncardiac surgery patients showed that high preoperative physiological burden was associated with up to twofold increases in postoperative mortality and elevated rates of myocardial infarction and venous thromboembolism. Epidemiological evidence further supports that anxiety and depression independently increase cardiovascular risk. Converging evidence suggests that preoperative psychological distress is a relevant perioperative cardiovascular risk factor. However, no randomized controlled trial has evaluated whether targeted preoperative stress reduction can decrease postoperative cardiovascular events, representing a critical gap warranting prospective investigation.

## 1. Introduction

Noncardiac surgeries are among the most common medical procedures performed worldwide [1]. An estimated 313 million surgical procedures are performed worldwide annually [2]. In the United States alone, perioperative death, myocardial infarction, or ischemic stroke occurs in approximately 1 in 33 surgical admissions among patients aged over 45 years, corresponding to over 150,000 annual perioperative cardiovascular events [3]. Myocardial infarction, cardiac arrest, congestive heart failure, and thromboembolic events remain leading causes of perioperative adverse outcomes [4]. While traditional risk stratification models have focused on established cardiovascular risk factors such as hypertension, diabetes, and coronary artery disease [3,5], growing evidence suggests that preoperative psychological and psychosocial factors may represent a group of underrecognized modifiable perioperative risk factors [4,6].

The biological mechanisms behind these associations are well known: chronic psychological distress activates the hypothalamic–pituitary–adrenal (HPA) axis and the sympathetic nervous system, causing a multitude of downstream effects including hypercoagulability, dyslipidemia, impaired glucose regulation, systemic inflammation, endothelial dysfunction, decreased heart rate variability, and increased arterial stiffness [7]. These pathophysiological processes are directly relevant to the mechanisms underlying perioperative cardiovascular injury.

For the purposes of this review, chronic stress refers to the cumulative physiological and psychological burden resulting from sustained or repeated exposure to stressors, as distinct from acute situational anxiety experienced in the immediate preoperative period. Self-reported questionnaires such as the K-6, HADS, GAD-7, and PHQ-9 measure recent psychological symptoms over a defined recall window and serve as proxies for, but not direct measures of, cumulative chronic stress exposure. Allostatic load quantifies multisystem physiological dysregulation that may result from chronic stress but also reflects aging, comorbidity, and socioeconomic factors. Throughout this review, the term “preoperative psychological distress” is used when referring to findings from single-timepoint questionnaire-based studies, while “chronic stress” is reserved for discussions of sustained exposure and its biological consequences. Where the evidence is extrapolated rather than directly demonstrated in noncardiac surgical populations, this distinction is noted.

Certain experts believe that our understanding of perioperative stress needs a fundamental revision. Manou-Stathopoulou et al. have argued that the conventional school of thought focusing solely on neuroendocrine activation following surgical trauma is inadequate for modern perioperative medicine, particularly in an era characterized by older and frailer patients with an often significant burden of comorbid psychological stress [8]. Chronic psychosocial stress and common cardiorespiratory comorbidities are now recognized as relevant modifiers of the neuroendocrine response to acute surgical injury, suggesting that the preoperative psychological state and patient’s level of chronic stress may alter the trajectory of surgical recovery [8].

Despite this evidence, preoperative psychological stress remains largely absent from current perioperative cardiovascular risk assessment protocols. No major guideline incorporates screening for chronic psychological stress as part of preoperative evaluation, and no randomized controlled trials have examined whether targeted preoperative stress reduction interventions can mitigate postoperative cardiovascular complications. This review aims to synthesize the current evidence linking preoperative chronic stress to postoperative cardiovascular dysfunction following noncardiac surgery, examining the epidemiological associations, underlying pathophysiological mechanisms, and potential implications for perioperative risk stratification and intervention.

## 2. Methods

A structured literature search was conducted in PubMed/MEDLINE, Embase, and Cochrane databases from inception through 31 May 2026. Key search terms included combinations of “chronic stress,” “psychological distress,” “allostatic load,” “cortisol,” “HPA axis,” “glucocorticoid resistance,” “noncardiac surgery,” “postoperative cardiovascular complications,” “myocardial injury after noncardiac surgery,” “MINS,” “venous thromboembolism,” “atrial fibrillation,” “endothelial dysfunction,” and “autonomic dysfunction.” Reference lists of systematic reviews were hand-searched to identify additional studies. No language restrictions were applied. Studies were selected based on relevance to the review question, with priority given to prospective clinical studies in noncardiac surgical populations, followed by systematic reviews and meta-analyses, mechanistic studies, and general-population epidemiological data. Because the terms used to describe stress vary widely in timeframe, measurement, and biological meaning, the stress-related constructs considered in this review, along with their definitions, common measurement instruments, relevant timeframes, and limitations in the perioperative setting, are summarized in Table 1. As direct evidence linking objectively measured preoperative chronic stress to cardiovascular-specific outcomes after noncardiac surgery remains limited, evidence from related domains including cardiac surgery, general cardiovascular epidemiology, pharmacological glucocorticoid exposure, and preclinical models were included where it informed the biological rationale. No formal quality assessment of individual studies was performed, consistent with the design of this publication, which is a narrative review.

## 3. Biomolecular Pathways of Chronic Stress

### 3.1. Hypothalamic–Pituitary–Adrenal (HPA) Axis and Stress

Cortisol plays a central role in the physiological stress response and in the regulation of metabolism, immune function, and cardiovascular homeostasis [9]. The hypothalamic–pituitary–adrenal (HPA) axis is activated in response to stressors through the hypothalamic release of corticotropin-releasing hormone (CRH). This hormone then stimulates the anterior pituitary gland to secrete adrenocorticotropic hormone (ACTH), which in turn drives cortisol synthesis and release from the adrenal cortex [9,10,11]. Under basal conditions, cortisol is secreted in a pulsatile, circadian pattern, with peak levels in the early morning and lowest values in the late evening [12,13].

Unbound, or free, cortisol is the biologically active form that binds to intracellular glucocorticoid receptors (GR) [14]. Upon binding, the GR migrates to the nucleus and regulates the transcription of target genes involved in metabolic, cardiovascular, and immune processes [10,14,15,16]. Besides direct genomic effects, glucocorticoids have an indirect effect on genes, resulting from the physical interaction between the monomeric glucocorticoid-GR complex and nuclear transcription factors such as NF-κB and AP-1, preventing the expression of genes encoding pro-inflammatory mediators [17,18]. Non-genomic effects occur more rapidly, within minutes, and are mediated through membrane-associated GR signaling [19,20]. Upon glucocorticoid binding, GR-associated proteins such as heat shock proteins (HSPs) and Src kinase are released, activating downstream signaling cascades [18,21]. Glucocorticoids can also increase cAMP production via Gαs-coupled signaling which is a more rapid pathway that contributes to approximately one-third of glucocorticoid gene expression modification effects [22].

Prolonged exposure to elevated cortisol levels leads to HPA axis dysregulation through several mechanisms. Chronic glucocorticoid excess downregulates GR density, producing glucocorticoid resistance [23,24]. At the molecular level, chronic stress alters GR phosphorylation, increases expression of the dominant-negative GRβ splice variant, and upregulates the co-chaperone FKBP5, all of which impair GR signaling and reduce cellular sensitivity to glucocorticoids [25]. At the same time, the normal pulsatile and circadian secretory patterns are lost and replaced by a flattened cortisol slope with elevated evening levels [9]. Such dysregulation encompasses more than the HPA axis, contributing to systemic inflammation, autoimmune susceptibility, and structural changes within the central nervous system, including the prefrontal cortex and hippocampus [26,27].

### 3.2. Molecular Basis of Glucocorticoid Resistance

Effects of chronic stress are not enforced only by increased cortisol levels; they alter glucocorticoid receptor (GR) signaling at multiple molecular levels, resulting in reduced anti-inflammatory responsiveness despite persistent glucocorticoid exposure which is a state termed glucocorticoid resistance (GCR) [25]. A systematic review across different species, including humans, confirmed that chronic stress is consistently associated with downregulation of cellular GR sensitivity and upregulation of pro-inflammatory biomarkers [24]. Alternative splicing of NR3C1 exon 9 generates two principal isoforms: GRα, the classic ligand-binding receptor mediating anti-inflammatory effects, and GRβ, which possesses a truncated ligand-binding domain which makes it incapable of binding glucocorticoids and functioning as an inhibitor of GRα through formation of transcriptionally inactive heterodimers [25,28]. Critically, GRβ specifically interferes with the ability of GRα to repress NF-κB, promoting a pro-inflammatory state even in the presence of glucocorticoids, while pro-inflammatory cytokines such as TNF-α selectively increase GRβ expression, creating a cycle of inflammation and resistance [25,28,29].

Post-translational modification of the GR through phosphorylation represents another critical mechanism of stress-induced receptor impairment. In human GRα, phosphorylation occurs primarily at serine residues 203, 211, and 226, and the S211/S226 phosphorylation ratio is critical in determining transcriptional activity [30]. The p38 mitogen-activated protein kinase (MAPK), activated by pro-inflammatory cytokines, increases GRα phosphorylation, reducing ligand-binding sensitivity and binding affinity for both glucocorticoid ligands and target DNA sequences [25,29]. Importantly, GRα phosphorylation also inhibits the interaction with the p65 subunit of NF-κB, leading to disinhibition of NF-κB-mediated inflammatory genes, thus promoting NF-κB-driven inflammation [25].

**Figure 1:**
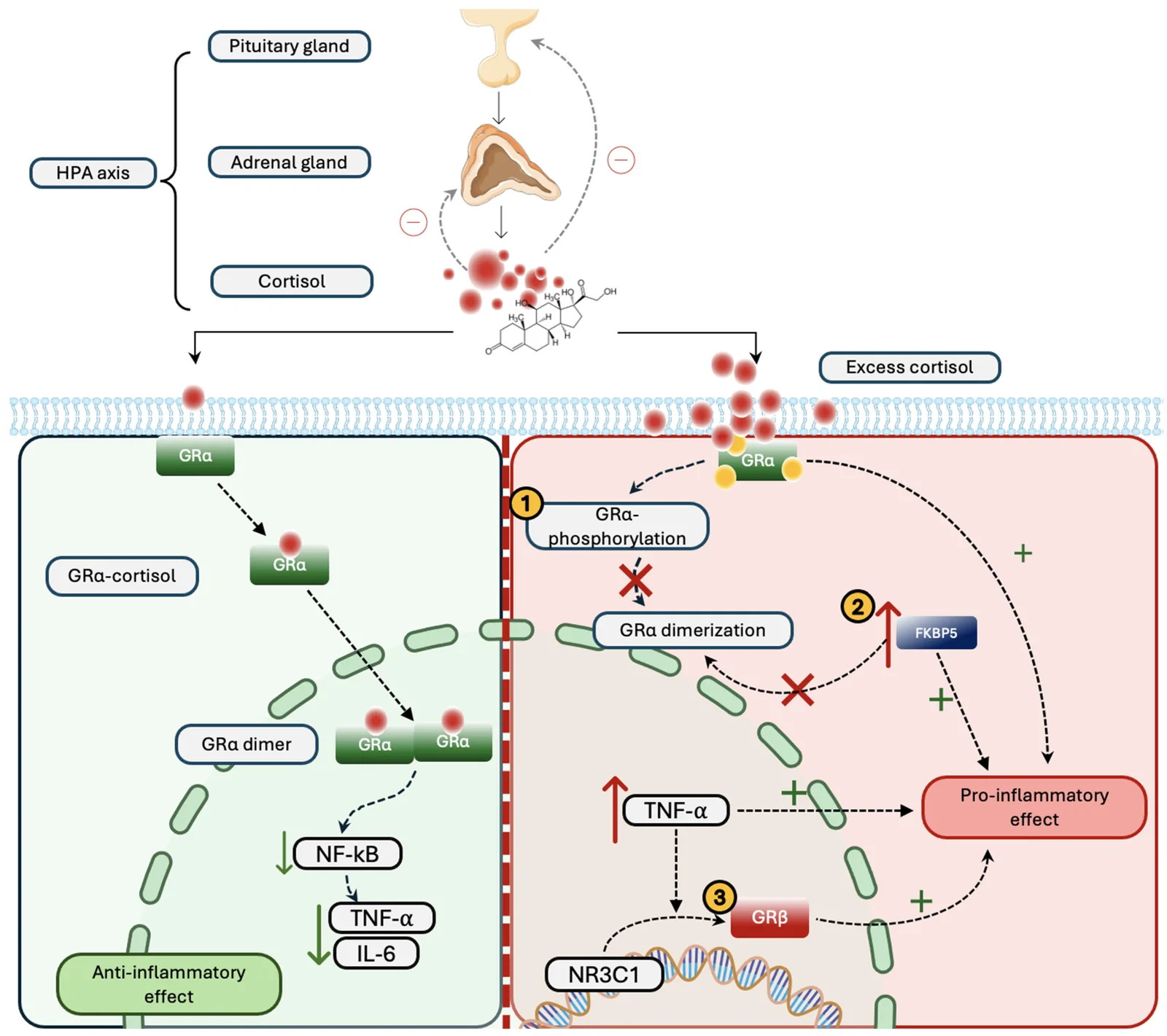
Molecular mechanisms of chronic stress-induced glucocorticoid resistance. **Left** panel: Under normal conditions, cortisol binds the glucocorticoid receptor alpha (GRα), which dimerizes, translocates to the nucleus, and suppresses NF-κB-driven transcription of pro-inflammatory cytokines (TNF-α, IL-6), producing an anti-inflammatory effect. **Right** panel: Under chronic stress with excess cortisol, three mechanisms impair this pathway: (1) p38 MAPK-mediated phosphorylation of GRα reduces ligand-binding affinity and inhibits interaction with NF-κB p65; (2) upregulation of the co-chaperone FKBP5 impairs GRα nuclear translocation and dimerization; (3) alternative splicing of NR3C1 increases expression of GRβ, which forms transcriptionally inactive heterodimers with GRα. TNF-α further upregulates GRβ expression, creating a self-reinforcing cycle. The net result is failure to suppress NF-κB, leading to sustained pro-inflammatory cytokine production. Encircled numerals (1–3) correspond to the three mechanisms of glucocorticoid resistance described above. Solid arrows indicate direct molecular steps or receptor translocation; dashed arrows indicate signaling or transcriptional regulation; curved arrows denote HPA-axis feedback. Upward (↑) and downward (↓) arrows indicate increased and decreased expression or activity, respectively. Red X symbols mark blocked pathways, green plus symbols indicate upregulation or promotion, and circled minus symbols (⊖) indicate negative feedback. Red dots represent cortisol molecules and yellow structures denote the membrane-associated GRα complex. Green shading denotes the physiological anti-inflammatory state and red shading the chronic-stress pro-inflammatory state Abbreviations: FKBP5, FK506-binding protein 5; GRα, glucocorticoid receptor alpha; GRβ, glucocorticoid receptor beta; HPA, hypothalamic–pituitary–adrenal; IL-6, interleukin-6; NF-κB, nuclear factor kappa-light-chain-enhancer of activated B cells; NR3C1, nuclear receptor subfamily 3 group C member 1; TNF-α, tumor necrosis factor alpha.

These multiple mechanisms produce a state of functional glucocorticoid resistance in which the anti-inflammatory and immunomodulatory actions of cortisol are progressively decreased despite elevated circulating glucocorticoid levels. The downstream consequence is a failure to control NF-κB-driven inflammation, resulting in dysregulated production of pro-inflammatory cytokines including IL-1β, TNF-α, and IL-6 [23,25] (Figure 1).

### 3.3. Chronic Stress and Endothelial Dysfunction

The vascular endothelium is considered a key site of chronic stress-mediated injury, with endothelial dysfunction representing one of the earliest detectable steps in atherogenesis [31,32]. Chronic stress alters endothelial function through converging glucocorticoid, catecholamine, and inflammatory pathways that reduce nitric oxide (NO) bioavailability and shift the endothelium toward a pro-inflammatory, vasoconstrictive phenotype [31].

At the molecular level, glucocorticoids impair endothelial function through multiple mechanisms. Cortisol directly downregulates endothelial nitric oxide synthase (eNOS) expression by decreasing eNOS mRNA stability and reducing the binding of transcription factor GATA at the eNOS promoter site [33,34]. Glucocorticoids also reduce the availability of the essential eNOS cofactor tetrahydrobiopterin (BH4), promoting eNOS uncoupling which switches the enzyme from producing protective NO to generating superoxide [35,36]. Simultaneously, glucocorticoids stimulate endothelin-1 (ET-1) production by promoting transcription of prepro-ET mRNA in vascular smooth muscle cells, creating a functional imbalance between vasodilation and vasoconstriction [36].

**Figure 2:**
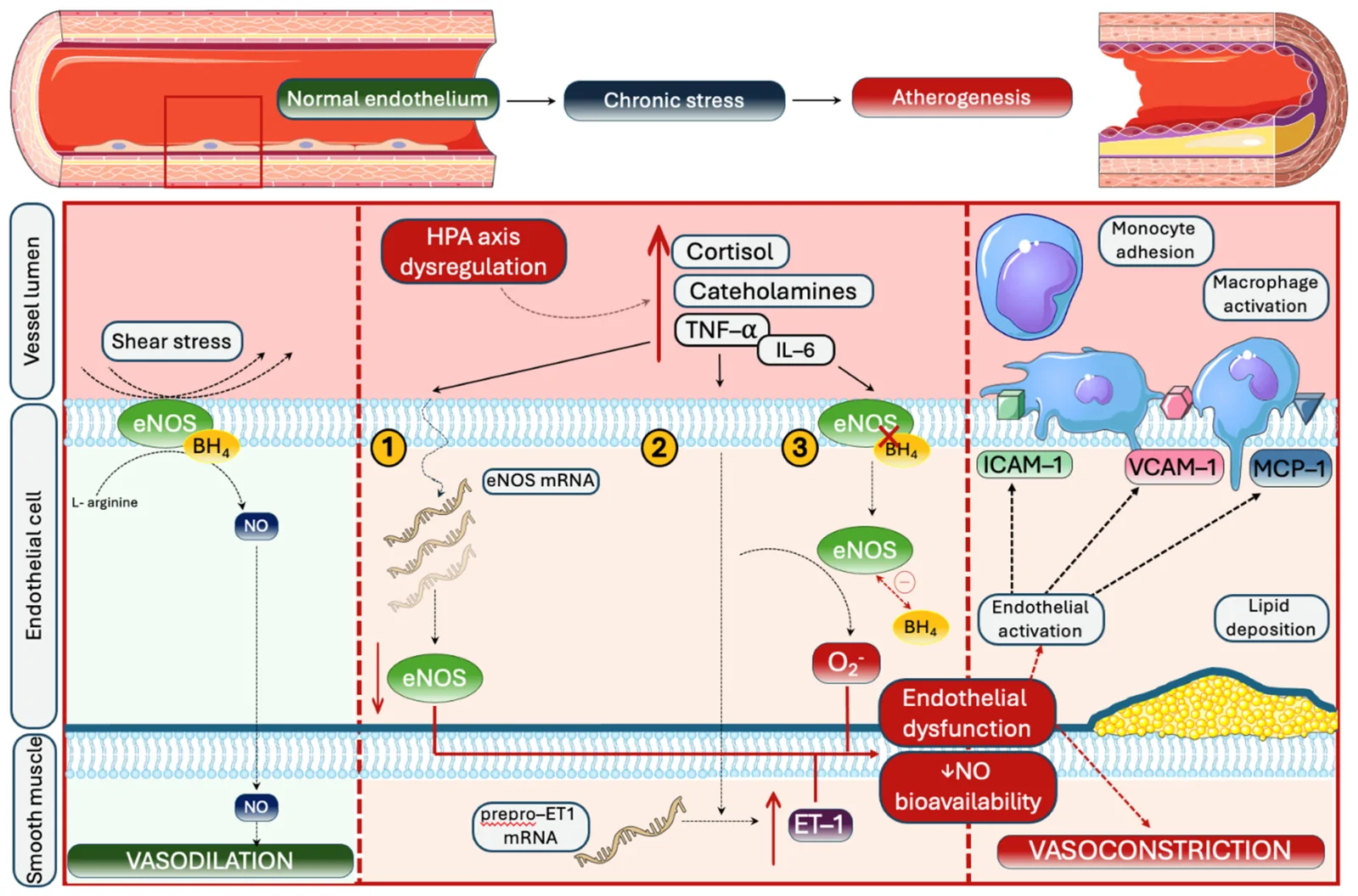
Molecular mechanisms of chronic stress-induced endothelial dysfunction and early atherogenesis. Left panel: Under normal conditions, endothelial nitric oxide synthase (eNOS), coupled with its cofactor tetrahydrobiopterin (BH_4_), converts L-arginine to nitric oxide (NO), which diffuses to vascular smooth muscle to promote vasodilation. Right panel: Chronic stress, through HPA axis dysregulation, elevates cortisol, catecholamines, and pro-inflammatory cytokines (TNF-α, IL-6), which impair endothelial function through three sequential mechanisms: (1) cortisol reduces eNOS mRNA stability and transcription factor GATA binding at the eNOS promoter; (2) glucocorticoids deplete BH_4_; (3) uncoupled eNOS generates superoxide (O_2_^−^) instead of NO, producing oxidative stress and endothelial dysfunction. Simultaneously, glucocorticoids stimulate prepro-endothelin-1 (ET-1) mRNA transcription in smooth muscle, shifting the balance toward vasoconstriction. Endothelial activation leads to upregulation of adhesion molecules (ICAM-1, VCAM-1, MCP-1), promoting monocyte adhesion, macrophage activation, and subendothelial lipid deposition. Encircled numerals (①–③) denote the three sequential mechanisms described above. Upward arrows (↑) indicate increased expression, concentration, or activity; downward arrows (↓) indicate decreased expression or bioavailability. Solid arrows indicate direct molecular steps or conversions; dashed arrows indicate signaling or transcriptional regulation. Red dashed arrows/lines denote pathological (chronic-stress) effects. Green shading denotes the normal vasodilatory state; red/pink shading denotes the chronic-stress vasoconstrictive and pro-atherogenic state. Abbreviations: BH_4_, tetrahydrobiopterin; eNOS, endothelial nitric oxide synthase; ET-1, endothelin-1; GATA, GATA-binding transcription factor; HPA, hypothalamic–pituitary–adrenal; ICAM-1, intercellular adhesion molecule-1; IL-6, interleukin-6; MCP-1, monocyte chemoattractant protein-1; NO, nitric oxide; O_2_^−^, superoxide; TNF-α, tumor necrosis factor alpha; VCAM-1, vascular cell adhesion molecule-1.

Beyond NO depletion, chronic stress promotes endothelial activation and vascular inflammation through upregulation of adhesion molecules. In a rabbit model, 16 weeks of unpredictable chronic stress significantly increased aortic expression of ICAM-1 and MCP-1, downregulated eNOS, and produced macrophage infiltration and subendothelial lipid accumulation [37]. Data from the Multi-Ethnic Study of Atherosclerosis confirmed that higher chronic stress was linked to lower rates of physiologic vasodilatation and higher circulating ICAM-1 levels [38]. Similarly, the AHA Scientific Statement on Psychological Health and CVD noted that women who developed PTSD exhibited larger increases in VCAM-1 relative to trauma-free controls, linking chronic psychological distress to endothelial activation in a prospective human cohort [7] (Figure 2).

### 3.4. Cardiovascular Effects of Glucocorticoids

The cardiovascular effects of glucocorticoid excess are most clearly demonstrated in Cushing’s syndrome, in which hypercortisolism produces hypertension, insulin resistance or diabetes, dyslipidemia, visceral obesity, hypercoagulability, and premature atherosclerosis [39,40]. Although Cushing’s syndrome is not equivalent to chronic psychological stress, it illustrates the end-organ effects of sustained cortisol elevation. Cushing’s syndrome carries an increased risk of both myocardial infarction and cardiac failure, with concentric left ventricular hypertrophy and impaired diastolic filling that develop independently of blood pressure, suggesting a direct glucocorticoid-mediated cardiac effect [40]. Consistently increased cortisol levels activate both the mineralocorticoid and the glucocorticoid receptors on cardiomyocytes, promoting myocardial fibrosis through increased responsiveness to angiotensin II and direct mineralocorticoid receptor activation [39,41].

At the vascular level, glucocorticoid excess impairs endothelial function through multiple mechanisms: inhibition of eNOS, depletion of the cofactor tetrahydrobiopterin, stimulation of endothelin-1 production, and generation of reactive oxygen species via NADPH oxidase, mitochondrial electron transport chain, and xanthine oxidase pathways [35,42,43]. Broadley et al. provided direct experimental evidence that cortisol mediates the acute vascular effects of psychological stress: pharmacological inhibition of cortisol production with metyrapone prevented mental stress-induced endothelial dysfunction in healthy volunteers [36]. Another study showed that even low-dose exogenous glucocorticoid use was associated with a significantly increased risk of atrial fibrillation, heart failure, myocardial infarction, and peripheral arterial disease in a dose-dependent fashion [44].

### 3.5. Chronic Stress and Immune Dysregulation

The immune system is primarily modulated by neuroendocrine pathways through the HPA axis and the sympathetic nervous system [45,46]. During acute stress, cortisol plays a role in limiting excessive inflammatory response activation through suppression of pro-inflammatory cytokines (IL-1β, IL-6, and TNF-α) and an increase in the anti-inflammatory cytokine IL-10 [47,48]. However, prolonged exposure to elevated cortisol levels promotes immunosuppression and immune dysregulation [45]. As described in Section 3.2, chronic glucocorticoid excess leads to glucocorticoid receptor resistance, affecting the normal anti-inflammatory feedback of cortisol and allowing a pro-inflammatory state [9,49]. Additionally, excess cortisol can bind to mineralocorticoid receptors, as previously described, with the effect of upregulating pro-inflammatory cytokine genes through activation of the NF-κB pathway and differentiation of macrophages toward a pro-inflammatory phenotype [50,51]. Glucocorticoids also impair T-cell proliferation, maturation, and activity, compromising adaptive immunity and increasing vulnerability to infections [45,46].

**Figure 3:**
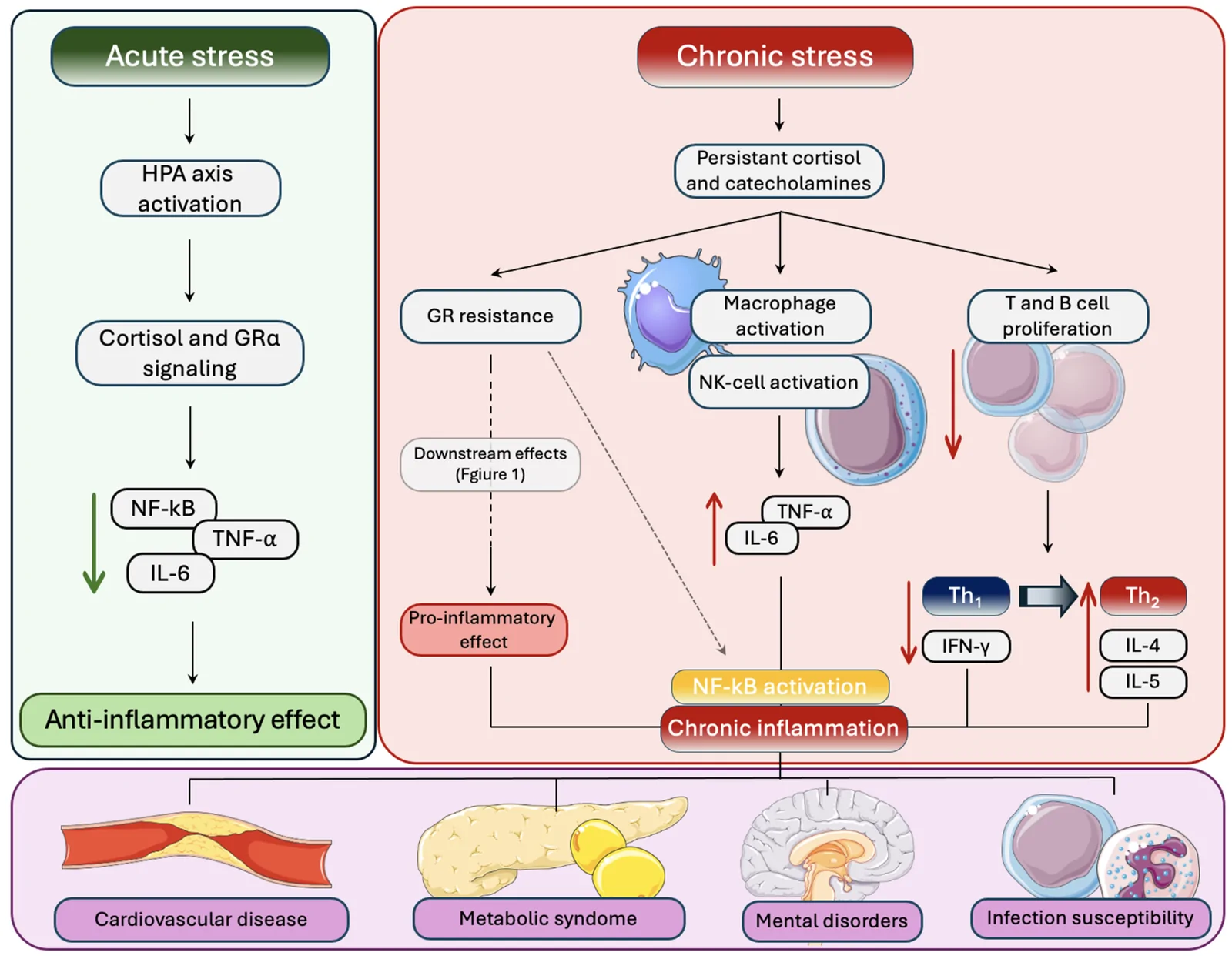
Clinical outcomes of chronic stress and immune dysregulation. **Left** panel: During acute stress, HPA axis activation produces cortisol, which binds GRα and suppresses NF-κB-driven pro-inflammatory cytokine production (TNF-α, IL-6), exerting an anti-inflammatory effect. **Right** panel: During chronic stress, persistent cortisol and catecholamine elevation leads to glucocorticoid receptor resistance (see Figure 1), macrophage activation, NK-cell activation, and altered T and B cell proliferation. Catecholamines acting via β-adrenergic receptors shift T-helper balance from Th1 (IFN-γ) toward Th2 (IL-4, IL-5) responses. Unopposed NF-κB activation drives chronic inflammation, contributing to downstream clinical consequences including cardiovascular disease, metabolic syndrome, mental disorders, and increased infection susceptibility. Upward arrows (↑) indicate increased and downward arrows (↓) decreased expression or activity; red arrows correspond to the chronic-stress state and the green downward arrow to cytokine suppression during the acute-stress anti-inflammatory response. Straight black arrows indicate direction of signaling or downstream effect. Green shading denotes the physiological anti-inflammatory state; red shading denotes the chronic-stress pro-inflammatory state. Abbreviations: GR, glucocorticoid receptor; GRα, glucocorticoid receptor alpha; HPA, hypothalamic–pituitary–adrenal; IFN-γ, interferon gamma; IL-4, interleukin-4; IL-5, interleukin-5; IL-6, interleukin-6; NF-κB, nuclear factor kappa-light-chain-enhancer of activated B cells; NK, natural killer; Th1, T-helper type 1; Th2, T-helper type 2; TNF-α, tumor necrosis factor alpha.

In parallel, exposure to different stressors activates the sympathetic nervous system, increasing circulating levels of catecholamines. Epinephrine and norepinephrine increase the innate immune cells’ activity, mainly natural killer (NK) cells and macrophages, while inhibiting adaptive immunity by modulating the function of regulatory T cells and B cells [52]. Catecholamines control the immune response during chronic stress via β-adrenergic receptors, favoring Th2-type responses over Th1 [47,53]. This shift increases susceptibility to viral infections and reduces tumor-cell surveillance, while the predominance of Th2 responses can exacerbate allergic and autoimmune pathology [54] (Figure 3).

## 4. Screening and Measurement

### 4.1. Screening Methods

The biggest challenge posed to currently available screening methods is how to appropriately approach preoperative distress and measure it precisely but also universally. Several scales have been developed with this specific question in mind. For the scope of this manuscript, screening methods looking specifically at acute preoperative stress (such as the APAIS and the STAI-State questionnaire) have been excluded.

The Hospital Anxiety and Depression Scale (HADS) is a 14-item self-report questionnaire developed specifically for detecting anxiety and depression in medically ill patients. It has two questionnaires with 7 items each, the HADS-Anxiety (HADS-A) and the HADS-Depression (HADS-D) subset [55,56]. The HADS has been validated in general surgery inpatients and used in the perioperative studies reviewed in this manuscript, including the Myoga et al. and Orri et al. cohorts.

The Kessler Psychological Distress Scale (K-6) is a 6-item questionnaire measuring nonspecific psychological distress, such as nervousness, hopelessness, restlessness, depression, effort, and worthlessness over 30 days preceding surgery [57]. The K-6 is free to use and takes approximately 2 min to administer [57]. Importantly, the K-6 was used in the VISION cohort study where each unit increase in score was independently associated with 30-day cardiovascular complications and 1-year mortality following noncardiac surgery, making it the only screening instrument with direct evidence linking preoperative distress scores to postoperative cardiovascular endpoints in this population [4].

The Generalized Anxiety Disorder 7-item scale (GAD-7) screens for generalized anxiety based on DSM-IV criteria, assessing symptoms over the two weeks preceding surgery [58]. The GAD-7 has demonstrated moderate-to-high diagnostic accuracy across multiple validation studies [59,60]. Its ultra-brief counterpart, the GAD-2, has acceptable sensitivity and specificity and is suitable for rapid preoperative screening [61].

The Patient Health Questionnaire-9 (PHQ-9) is a 9-item screening measure targeting major depressive disorder symptoms over the two weeks prior to surgery [58]. The PHQ-2 offers a 2-item ultra-brief alternative with good discriminant validity, making it suitable for rapid screening in preoperative clinics [61]. The PHQ-9 and GAD-7 were used together in the China Surgery and Anesthesia Cohort, where machine learning-based grouping of patients identified distinct psychological patterns associated with adverse surgical outcomes [62].

Notably, while these instruments capture clinically meaningful psychological distress, none was specifically designed to measure cumulative chronic stress exposure over months or years, a limitation addressed by biomarker-based approaches such as allostatic load, discussed in Section 4.2.

### 4.2. Stress and Allostatic Load

Allostatic load (AL) is a biomarker-based method that measures cumulative physiological burden resulting from sustained activation of stress-response systems across cardiovascular, metabolic, and immune domains [46,63,64,65]. Originally conceptualized by McEwen as the biological “price of adaptation,” AL captures multisystem physiological dysregulation through a composite of ten biomarkers, offering an objective addition to the self-reported psychological instruments discussed above [63,65]. A systematic review encompassing 267 original investigations confirmed that AL is consistently associated with poorer health outcomes across a variety of clinical settings [64]. In the noncardiac surgical context, emerging evidence demonstrates that elevated preoperative AL significantly worsens postoperative outcomes, including cardiovascular complications. Khalil et al. examined patients undergoing hepatopancreatobiliary cancer surgery and found that high AL was associated with an increased risk of myocardial infarction, thromboembolic events and 30-day mortality compared to patients with low AL [66]. Similar findings were reported by the same author group when looking at patients undergoing colorectal cancer surgery [67]. Chen et al. examined patients undergoing breast cancer surgery and found that high allostatic load was associated with a 29% increased odds of 30-day postoperative complications [68]. Notably, the AL score outperformed individual biomarkers in predicting complications, reinforcing the concept that cumulative multisystem dysregulation rather than any single physiological derangement captures the clinically relevant burden of chronic stress.

Importantly, AL is not specific to chronic psychological stress. Its components, including blood pressure, HbA1c, HDL cholesterol, waist-to-height ratio, CRP, and resting heart rate, substantially overlap with established cardiovascular risk factors and comorbidities already captured in standard perioperative risk assessment. AL is strongly influenced by age, with age being the strongest predictor of high AL in population studies [69]. AL also increases with lower socioeconomic status, minority race/ethnicity, obesity, and chronic disease burden independently of psychological stress exposure [70,71].

These studies reinforce the concept that cumulative multisystem physiological dysregulation, which may in part reflect chronic stress, translates into measurably worse surgical outcomes with the cardiovascular system appearing particularly vulnerable to the effects of sustained allostatic overload in the perioperative period [66,67]. Importantly, AL can be derived from routinely collected laboratory and clinical data, making it a potentially scalable preoperative screening tool that can help identify patients at higher risk for postoperative cardiovascular complications.

### 4.3. Biomarkers of Chronic Stress

Cortisol remains the most widely known and commonly used stress biomarker, but how it is measured fundamentally determines what it reflects. Serum and salivary cortisol capture a single timepoint and are subject to diurnal variation, pulsatile secretion, and acute situational influences, making them unreliable indicators of chronic stress exposure [72]. One surrogate measurement for accumulation of chronic stress over time that has been extensively researched is the hair cortisol concentration. Hair cortisol concentration has emerged as the most promising neuroendocrine biomarker of chronic stress, as each 1 cm hair segment approximates one month of cumulative cortisol production, enabling retrospective assessment of HPA axis activity over 3–6 months from a single sample [73,74]. A meta-analysis by Kuckuck et al. confirmed a significant association between higher hair cortisol concentration and cardiovascular disease largely independent of standard risk factors [19]. In a large Swedish population-based study, elevated hair cortisol concentration was significantly associated with a history of myocardial infarction, type 2 diabetes, atrial fibrillation, and bypass surgery, as well as with inflammatory markers including leukocytes and high-sensitivity CRP [75]. The measurement characteristics, advantages, limitations, and potential perioperative applications of the stress-related biomarkers discussed in this section are summarized in Table 2. 

One autonomic biomarker that is consistently affected and reduced by chronic stress is heart rate variability. As discussed in Section 5.3, May et al. demonstrated that acquired loss of cardiac vagal activity was significantly associated with postoperative myocardial injury in noncardiac surgery patients [76]. A recent systematic review by Pan et al. confirmed that perioperative autonomic nervous system imbalance which is characterized by sympathetic hyperactivation and parasympathetic withdrawal is associated with adverse surgical outcomes including cardiovascular complications, infections, and neurocognitive decline [77]. Resting heart rate, a simpler surrogate of autonomic tone, was identified in McCrory et al. as one of five core allostatic load biomarkers that reliably predicted mortality [78].

CRP was identified in the McCrory et al. consensus allostatic load definition as one of five biomarkers (alongside resting heart rate, HDL cholesterol, waist-to-height ratio, and HbA1c) that reliably and consistently predicted health outcomes across 13 cohort studies [78]. This 5-item allostatic load index predicted mortality as well as or better than more elaborate biomarker panels, suggesting that a brief, routinely available set of markers may be sufficient for preoperative chronic stress screening [78]. While these biomarkers are routinely available, they are influenced by multiple clinical conditions beyond psychological stress, including obesity, diabetes, infection, and cardiovascular disease.

Several novel approaches are under investigation for quantifying chronic stress at the molecular level. DNA methylation-based algorithms (GrimAge, DunedinPACE) estimate biological age, which accelerates under chronic psychosocial stress. Suglia et al. demonstrated that cumulative life stress predicted accelerated epigenetic aging. Major surgery itself induces acute epigenetic changes in immune response genes, raising the question of whether preoperative epigenetic age acceleration adds to the risk of surgical complications [79,80].

A critical limitation shared by all biomarkers is their specificity. Hair cortisol concentration is influenced by BMI, hair characteristics, and oral contraceptive use, and a comprehensive meta-analysis found no consistent association between HCC and self-reported perceived stress or depressive symptoms [74]. A recent multiverse analysis confirmed that biological and behavioral confounders were stronger determinants of hair glucocorticoids than most psychosocial variables in young adults [81]. CRP is a nonspecific marker of systemic inflammation elevated by infection, obesity, autoimmune disease, malignancy, and tissue injury. Resting heart rate and HRV are influenced by age, fitness level, medications, and cardiopulmonary disease. In conclusion, none of these biomarkers has been validated as a stand-alone preoperative measure of chronic psychological stress.

## 5. From Chronic Stress to Preoperative Cardiovascular Injury

The following sections examine how the biological consequences of chronic stress described in Section 3 intersect with the mechanisms of perioperative cardiovascular injury. It is important to note that this connection is largely inferential. The mechanistic pathways of chronic stress and the pathophysiology of perioperative myocardial injury, arrhythmogenesis, and venous thromboembolism are each well characterized independently, but few studies have directly demonstrated that preoperative chronic stress amplifies these specific perioperative triggers in noncardiac surgery patients.

### 5.1. Myocardial Injury After Noncardiac Surgery (MINS) and Perioperative Myocardial Infarction

Myocardial injury after noncardiac surgery (MINS) is defined as an elevated postoperative cardiac troponin concentration exceeding the 99th percentile of the upper reference limit, of presumed ischemic origin, with or without concomitant ischemic symptoms or signs [82]. MINS encompasses both type 1 MI (atherothrombotic plaque disruption) and type 2 MI (supply–demand mismatch), as well as asymptomatic ischemic myocardial injury, because surgical patients may be unable to report symptoms due to anesthesia, analgesia, or distracting surgical pain [3]. MINS is distinct from nonischemic causes of postoperative troponin elevation such as pulmonary embolism, sepsis, and stress cardiomyopathy, which are excluded from the diagnosis [3].

Perioperative myocardial injury occurs in approximately 20% of patients undergoing noncardiac surgery and is associated with 30-day mortality of approximately 10% [3]. The AHA Scientific Statement on MINS identifies the key perioperative triggers: surges in catecholamines, cortisol, and inflammatory cytokines from anesthesia and surgical trauma lead to hemodynamic fluctuations, tachycardia-induced supply–demand mismatch, increased coronary shear stress with plaque destabilization, and a prothrombotic state driven by platelet activation and hypercoagulability [82]. Chronic stress may prime each of these pathways through the mechanisms described in Section 3, although direct evidence that preoperative chronic stress amplifies these specific triggers in noncardiac surgery patients remains limited. As discussed in Section 3.1, chronic HPA axis dysregulation produces sustained sympathetic activation and elevated basal catecholamine levels, which lower the threshold for perioperative tachycardia and hypertension, both of which are direct drivers of myocardial oxygen supply–demand mismatch (type 2 MI) [82,83]. Simultaneously, the chronic inflammatory state described in Section 3.5. accelerates atherosclerotic plaque progression and reduces plaque stability through increased macrophage infiltration, thinning of the fibrous cap, and neovascularization [84]. When superimposed surgical stress further destabilizes these vulnerable plaques, they become more susceptible to rupture, precipitating type 1 perioperative MI [82,84]. Notably, in a non-surgical, general population study, Lazzarino et al. demonstrated that heightened cortisol response to mental stress is associated with detectable circulating high-sensitivity cardiac troponin T even in healthy adults without cardiovascular disease, suggesting that cortisol may contribute to cardiomyocyte injury through mechanisms beyond atherosclerosis [85]. Bollen Pinto and Ackland have further proposed that perioperative troponin elevation may reflect a nonspecific marker of cardiomyocyte stress rather than ischemia alone, with systemic inflammation, adrenergic stress, and autonomic dysfunction acting as converging triggers in a multi-hit model [86].

The reported incidence of MINS varies with the troponin assay, surveillance strategy, and patient population. In the VISION study, MINS occurred in 18% of patients; however, when conventional fourth-generation troponin was used, incidence was 8% [82]. Thirty-day mortality associated with MINS is approximately 10% overall but is strongly proportional to peak troponin concentration (17% in the highest quartile vs. 1% in the lowest) [3]. Importantly, 80–90% of MINS events are asymptomatic and would go undetected without routine postoperative troponin surveillance [87].

### 5.2. Hypercoagulability and Venous Thromboembolism

Both acute and chronic stress are associated with increased risk of atherosclerotic cardiovascular disease and venous thromboembolism through a hypercoagulable state [88]. The proposed mechanisms involve catecholamines and cortisol, which have been shown to activate platelets, upregulate coagulation factors, and impair fibrinolysis [88,89]. Consistent with the allostatic load concept, acute stress-induced hypercoagulability is initially an adaptive response to protect against bleeding in fight-or-flight situations, but becomes maladaptive under chronic stress, producing a sustained low-grade prothrombotic state [88,90]. In the perioperative setting, other factors further enhance this chronic prothrombotic baseline, including immobilization, tissue injury, and catecholamine surges, substantially increasing the risk of both arterial thrombotic events and venous thromboembolism [82,88]. Recent experimental work by Fall et al. has provided the first in vivo evidence that acute psychological stress triggers systemic free radical formation in a non-surgical population, identifying oxidative stress as a mechanistic link between psychological stress and hypercoagulability [91].

### 5.3. Autonomic Dysfunction and Arrhythmogenesis

Chronic stress produces sustained sympathovagal imbalance characterized by sympathetic hyperactivation and parasympathetic withdrawal which manifests in a multitude of ways such as reduced heart rate variability (HRV) and impaired baroreflex sensitivity [77,92,93]. May et al. demonstrated in a prospective cohort of 189 noncardiac surgery patients that acquired loss of cardiac vagal activity was significantly associated with postoperative myocardial injury (troponin ≥ 15 ng/L within 48 h), even in patients classified as low-risk by the Revised Cardiac Risk Index [76]. The AHA Scientific Statement on MINS similarly identifies impaired heart rate recovery (≤12 BPM decrease in the first minute after exercise), a marker of parasympathetic dysfunction, as an independent predictor of MINS [82]. At the cellular level, chronic adrenergic agonism disrupts cardiomyocyte calcium dynamics, impairs the circadian rhythm of cardiac cells, and promotes fibroblast activation, all of which predispose to arrhythmias [92]. New-onset postoperative atrial fibrillation (POAF) occurs in approximately 3–16% of noncardiac surgery patients depending on surgery type and is associated with a 2-fold increased risk of stroke (HR 2.00, 95% CI 1.70–2.35) and significantly increased short- and long-term mortality [3,94,95]. The pathophysiology of POAF involves gap junction uncoupling triggered by inflammation, ischemia, and autonomic imbalance, all of which may be amplified in patients with preexisting chronic stress-induced autonomic dysfunction [94].

### 5.4. Endothelial Dysfunction and Coronary Microvascular Disease

Stress hormones, including glucocorticoids, pro-inflammatory cytokines, and endothelin-1, are crucial components in the development of endothelial dysfunction through downregulation of endothelial nitric oxide (NO) production and impaired vasodilation [96]. The degree of mental stress-induced endothelial dysfunction correlates with changes in systemic vascular resistance, which is directly associated with changes in left ventricular ejection fraction and myocardial ischemia [96]. In the coronary microvasculature, impaired endothelium-dependent vasodilation leads to inadequate coronary perfusion, while uninhibited platelet aggregation and thrombosis can precipitate myocardial ischemia or infarction [96]. Dai et al. demonstrated that stress-related amygdalar activity promotes cardiovascular events through increased bone-marrow leukopoietic activity, which in turn promotes coronary inflammation and development of high-risk coronary plaque features [97].

## 6. Clinical Evidence

### Preoperative Anxiety, Depression, and Psychological Distress

Although anxiety, depression, and chronic stress frequently co-occur and share neuroendocrine pathways, they represent distinct constructs with different associations with cardiovascular outcomes. This distinction is clinically important because the perioperative studies reviewed in this manuscript used instruments that capture composite psychological distress without separating these components. The noncardiac perioperative studies assessing preoperative stress or distress and postoperative outcomes are summarized in Table 3, allostatic load studies in surgical cohorts in Table 4, and the supporting general-population epidemiology and cardiac surgery evidence in Table 5. 

Anxiety is an established independent risk factor for cardiovascular disease in the general population. A meta-analysis of 46 cohort studies including a total of 2,017,276 participants conducted by Emdin et al. found that anxiety was associated with significantly elevated risks of cardiovascular mortality (RR 1.41), coronary heart disease (RR 1.41), stroke (RR 1.71), and heart failure (RR 1.35) [98]. A UK Biobank prospective cohort study confirmed that anxiety disorder alone was associated with a 72% increased risk of CVD and that this risk was additive with depression, meaning that patients with both conditions had the highest risk (HR 2.89) [99]. In the perioperative setting, however, the evidence for anxiety specifically predicting cardiovascular complications is less consistent. Kassahun et al. found that preoperative anxiety measured by the STAI (≥40) was associated with morbidity only in the subgroup undergoing complex surgery but not in the overall cohort [100]. The VISION cohort sensitivity analysis similarly found that anxiety did not independently predict 1-year mortality [4]. A 2026 two-center study by Kraft et al. in colorectal surgery patients found that higher HADS scores were an independent risk factor for clinically relevant postoperative complications, though this reflected combined anxiety and depression rather than anxiety alone [101].

Depression has a more consistent association with cardiovascular outcomes. Penninx demonstrated that major depressive disorder increases the risk of cardiovascular morbidity and mortality by approximately 80%, mediated through both behavioral pathways (smoking, physical inactivity, medication non-adherence) and pathophysiological mechanisms (autonomic dysfunction, HPA axis dysregulation, metabolic disturbances, and immuno-inflammatory activation) [102]. The AHA has formally recognized depression as a risk factor for poor prognosis in patients with acute coronary syndrome [103]. In the perioperative context, the VISION cohort sensitivity analysis specifically identified depressive symptoms as the driver of the 1-year mortality association, while anxiety was not significant [4]. Orri et al. prospectively studied 591 patients undergoing liver resection and found that 24% were depressed preoperatively when measured with the HADS questionnaire; while depression was not independently correlated with morbidity or mortality in this cohort, it was a strong independent predictor of prolonged length of stay and was the primary factor explaining the gap between surgeon-anticipated and actual length of stay [104]. A systematic review and meta-analysis of preoperative depression in older surgical patients (≥65 years) found a pooled prevalence of 23% and a twofold increased risk of postoperative delirium [105]. In a large Medicare analysis of nearly 1.9 million surgical patients, Paredes et al. found that preexisting anxiety/depression was associated with 44% higher odds of surgical complications and 87% higher odds of 30-day readmission [106].

Composite psychological distress measured without separating anxiety from depression has also been independently associated with adverse perioperative outcomes. In a prospective study of 721 surgical patients, Myoga et al. demonstrated that preoperative psychological stress was independently associated with postoperative complications even after adjustment for age, alcohol use, tobacco use, and anesthesia duration; notably, neither drinking nor smoking habits were independently associated with complications in this cohort [6]. Data from the VISION cohort study reinforced that higher levels of preoperative psychological distress are independently associated with both 30-day cardiovascular complications (myocardial infarction, cardiac arrest, DVT/PE, and congestive heart failure) and 1-year mortality after noncardiac surgery [4]. In the largest cohort to date, Hou et al. analyzed 10,932 patients from the China Surgery and Anesthesia Cohort (CSAC) and applied unsupervised machine learning to classify patients into six distinct psychological distress patterns based on PHQ-9 and GAD-7 item-level data [62]. The “nervousness” pattern was associated with a 33% increased risk of postoperative complications, while the “highly combined symptoms” pattern exhibited the strongest associations across multiple outcomes. Notably, conventional cutoff-based classification of anxiety and depression showed no significant association with complications in the same cohort, suggesting that the heterogeneity of psychological distress presentations may be inadequately captured by simple screening thresholds [62].

Chronic stress is conceptually distinct from both anxiety and depression. While anxiety and depression are clinical diagnoses or symptom states typically measured at a single timepoint, chronic stress refers to the cumulative physiological burden of sustained or repeated exposure to psychosocial stressors over weeks, months, or years. As described in Section 3 and Section 4.2, chronic stress produces measurable multisystem dysregulation including HPA axis disruption, glucocorticoid resistance, sympathovagal imbalance, and systemic inflammation. A patient may have significant chronic occupational or psychosocial stress without meeting criteria for clinical anxiety or depression yet still carry the pathophysiological burden that increases perioperative cardiovascular risk. Conversely, anxiety and depression may serve as both markers and mediators of chronic stress. This overlap explains why instruments measuring psychological distress (K-6, HADS) capture some but not all of the chronic stress signal, and why biomarker-based approaches such as allostatic load may complement self-report instruments by measuring the cumulative physiological burden. Tawakol et al. demonstrated that resting metabolic activity within the amygdala independently predicted subsequent cardiovascular events [107]. A subsequent meta-analysis confirmed that heightened stress-related neural activity independently predicted composite adverse cardiovascular events [108]. Ronaldson et al. showed that a flatter presurgical diurnal cortisol slope predicted major adverse cardiac events and death in the years following coronary artery bypass grafting; although this study examined cardiac surgery patients, the cortisol-cardiovascular mechanism is directly applicable to the noncardiac surgical context [109]. A recent study by Abohashem et al. using PET/CT imaging, heart rate variability, and CRP demonstrated that both depression and anxiety are independently associated with increased MACE risk, mediated by heightened stress-related neural activity, autonomic dysfunction, and systemic inflammation [110].

**Table 3:** Noncardiac perioperative studies assessing preoperative stress/distress and postoperative outcomes.

Study	Design	*N*	Population	Instrument	Primary Outcome	Adjusted Effect Estimate	Key Covariates	Key Findings
*El-Gabalawy et al. 2025 [4]*	PC	938	Mixed noncardiac surgery	K6	30-day complications; 1-year mortality	30-day complications: aOR 1.12 (1.02–1.22) per K-6 unit; 1-year mortality: aOR 1.09 (1.02–1.18) per K-6 unit	Age, ethnicity, sex, surgery type, preoperative medical morbidity, smoking	Higher K6 is associated with 30-day complications and 1-year mortality driven by depression.
*Myoga et al. 2021 [6]*	PC	721	Mixed noncardiac surgery	K6; HADS	Postoperative complications (composite)	Psychological stress: *p* = 0.041 in multivariable model (OR not reported)	Age, alcohol use, tobacco use, anesthesia duration	Psychological stress independently associated with complications.
*Kassahun et al. 2022 [100]*	PC	400	Major general surgery	STAI	Morbidity and mortality	Subgroup (complex surgery, *n* = 221): aOR 2.12 (1.14–3.96) for morbidity	Age, sex, ASA, BMI, comorbidities, surgery type	Overall: no significant difference. Subgroup (complex surgery): anxiety associated with complications. No mortality difference.
*Hou et al. 2025 [62]*	PC	10,932	Mixed noncardiac surgery	PHQ-9, GAD-7, ISI	Complications, LOS, cognitive impairment, life satisfaction	“Nervousness” pattern: 33% increased risk of complications (exact OR/CI not reported)	Machine learning-derived clusters	“Nervousness” pattern associated with complications; “highly combined symptoms” have strongest associations across outcomes.
*Orri et al. 2015 [104]*	PC	591	Liver resection	HADS	Morbidity/ mortality, LOS	Depression associated with prolonged LOS: aHR 0.65 (0.50–0.83)	Age, sex, ASA, extent of resection, associated procedures	Depression independent predictor of prolonged LOS. Not associated with morbidity/mortality.
*Kraft et al. 2026 [101]*	PC	161	Colorectal surgery (CRC + IBD)	HADS	Clavien-Dindo ≥ IIIa complications	HADS score: aOR 1.07 (1.01–1.13) per unit	Hemoglobin, IBD, smoking	Higher HADS independently predicted complications.
*Paredes et al. 2020 [106]*	RC	1,900,000	Medicare surgical beneficiaries	ICD-coded diagnoses	Complications, readmission	Complications: aOR 1.44; 30-day readmission: aOR 1.87	Demographics, comorbidities, procedure type	Anxiety/depression: complications OR 1.44; 30-day readmission OR 1.87.

Abbreviations: aOR, adjusted odds ratio; ASA, American Society of Anesthesiologists physical status; BMI, body mass index; CI, confidence interval; CRC, colorectal cancer; GAD-7, Generalized Anxiety Disorder 7-item scale; HADS, Hospital Anxiety and Depression Scale; IBD, inflammatory bowel disease; ICD, International Classification of Diseases; ISI, Insomnia Severity Index; K-6, Kessler Psychological Distress Scale (6-item); LOS, length of stay; ns, not significant; OR, odds ratio; PC, prospective cohort; PHQ-9, Patient Health Questionnaire (9-item); RC, retrospective cohort; STAI, State-Trait Anxiety Inventory.

**Table 4:** Allostatic load studies in surgical cohorts.

Study	Design	*N*	Population	Instrument	Primary Outcome	Adjusted Effect Estimate	Key Covariates	Key Findings
*Khalil et al. 2026 [66]*	RC	34,253	HPB cancer surgery	Allostatic load (10 biomarkers)	Complications, FTR, 30-day mortality	CD grade IV complications: aOR 1.44 (1.36–1.54); FTR: aOR 1.85 (1.60–2.13); 30-day mortality: aOR 1.92 (1.70–2.19)	CCI, SVI, age, sex	High AL associated with failure to rescue, 30-day mortality, MI and VTE.
*Khalil et al. 2025 [67]*	RC	40,520	Colorectal cancer surgery	Allostatic load (10 biomarkers)	Complications, LOS, 30-day mortality	Complications: aOR 1.48 (1.38–1.58); extended LOS: aOR 1.79 (1.67–1.90); 30-day mortality: aOR 2.13 (1.90–2.37)	CCI, SVI, age, sex	High AL associated with complications, extended length of stay, 30-day mortality, MI, DVT/PE.
*Chen et al. 2024 [68]*	RC	4459	Breast cancer surgery	Allostatic load (10 biomarkers)	30-day postoperative complications	High AL: aOR 1.29 (1.01–1.63); per 1-point AL increase: aOR 1.08 (1.02–1.16); per quartile increase: aOR 1.13 (1.01–1.26)	Age, race, insurance, comorbidities, stage, surgery type	High AL is associated with complications; each 1-point AL increases 8% higher odds.

Abbreviations: AL, allostatic load; aOR, adjusted odds ratio; CCI, Charlson Comorbidity Index; CD, Clavien-Dindo; FTR, failure to rescue; HPB, hepatopancreatobiliary; LOS, length of stay; RC, retrospective cohort; SVI, Social Vulnerability Index.

**Table 5:** General population epidemiology and cardiac surgery.

Study	Design	*N*	Population	Instrument	Primary Outcome	Adjusted Effect Estimate	Key Covariates	Key Findings
*Emdin et al. 2016 [98]*	MA	2,017,276	General population	Various anxiety measures	CV mortality, CHD, stroke, HF	CV mortality: RR 1.41 (1.13–1.76); CHD: RR 1.41 (1.23–1.61); stroke: RR 1.71 (1.18–2.50); HF: RR 1.35 (1.11–1.64)	Varied by study	Anxiety associated with CV mortality, CHD, stroke, HF.
*Nakada et al. 2023 [99]*	PC	~502,000	General population (UK Biobank)	ICD-coded diagnoses	Incident CVD	Anxiety alone: HR 1.72; depression alone: HR 1.76; both: HR 2.89	Demographics, lifestyle, comorbidities	Anxiety alone HR 1.72; depression alone HR 1.76; both HR 2.89.
*Ronaldson et al. 2015 [109]*	PC	250	CABG patients	Diurnal cortisol slope	MACE and death	Steeper slope associated with reduced risk: HR 0.73 (0.56–0.96)	EuroSCORE, chronic illness burden, cardiopulmonary bypass	Flatter cortisol slope predicted MACE/death.
*Abohashem et al. 2026 [110]*	RC	85,551	General population (MGB Biobank)	PET/CT, HRV, CRP	MACE	Depression: HR 1.24 (1.14–1.34); concurrent anxiety: HR 1.35 (1.23–1.49)	Demographics, lifestyle, CV risk factors, socioeconomic factors	Depression and anxiety associated with MACE mediated by amygdalar activity, autonomic dysfunction, inflammation

Abbreviations: CABG, coronary artery bypass grafting; CHD, coronary heart disease; CRP, C-reactive protein; CV, cardiovascular; CVD, cardiovascular disease; HF, heart failure; HR, hazard ratio; HRV, heart rate variability; ICD, International Classification of Diseases; MA, meta-analysis; MACE, major adverse cardiovascular events; MGB, Mass General Brigham; PC, prospective cohort; PET/CT, positron emission tomography/computed tomography; RC, retrospective cohort; RR, relative risk.

## 7. Clinical Implications and Future Directions

Current perioperative cardiovascular risk assessment tools do not incorporate psychological stress, anxiety, or depression as predictive variables [5]. While the evidence reviewed here supports mechanistic plausibility and observational associations, it does not establish that preoperative stress assessment is ready for routine perioperative cardiovascular risk stratification. Nevertheless, recognition and appropriate management of clinically significant preoperative anxiety and depression is supported by general medical evidence and may be considered part of comprehensive preoperative assessment. The AHA has formally recognized depression as a risk factor for poor prognosis in patients with acute coronary syndrome, and the 2024 AHA/ACC Perioperative Guideline acknowledges the importance of patient-centered preoperative evaluation [3]. Identification of severe anxiety or depression during preoperative assessment may prompt referral for psychiatric evaluation or optimization of existing psychotropic medications [111].

Several approaches to preoperative stress management show promise; however, none have been specifically evaluated for reducing postoperative cardiovascular risk. Preoperative psychological interventions, including cognitive behavioral therapy (CBT), supportive psychotherapy, and acceptance and commitment therapy (ACT), collectively referred to as psychological prehabilitation, have been shown to reduce postoperative length of stay, pain, anxiety, and depression [112,113]. Multimodal prehabilitation combining exercise, nutritional optimization, and psychological support has also emerged as a promising strategy. However, Levett and Grimmett noted that although psychological factors consistently predict worse surgical outcomes, there is currently insufficient evidence to determine which psychological interventions are most effective or whether they independently improve physiological outcomes [114].

Ultimately, this review identified several important knowledge gaps that warrant prospective investigation. Future studies should determine whether validated measures of chronic stress (e.g., allostatic load, hair cortisol concentration, or composite psychometric instruments) improve cardiovascular risk discrimination when incorporated into established perioperative risk models such as the Revised Cardiac Risk Index (RCRI). In addition, no randomized controlled trial has evaluated whether targeted preoperative stress reduction through psychological prehabilitation, pharmacological treatment of anxiety or depression, exercise-based interventions, or multimodal prehabilitation programs can reduce postoperative cardiovascular events following noncardiac surgery.

## 8. Limitations

The central limitation of this review is that it addresses a question without abundant direct evidence from prospective studies or trials. While the cardiovascular consequences of chronic stress and the mechanisms of perioperative cardiovascular injury are well characterized independently, few studies have examined whether patients with documented preoperative chronic stress experience more postoperative cardiovascular events after noncardiac surgery. The available perioperative studies used heterogeneous instruments (K6, HADS, STAI, APAIS, Distress Thermometer) that predominantly capture acute psychological distress at a single preoperative time point rather than chronic stress exposure over months or years. No prospective study has measured a validated chronic stress biomarker preoperatively in a noncardiac surgery cohort and tracked cardiovascular-specific endpoints as the primary outcome. Furthermore, no randomized controlled trial has tested whether targeted preoperative stress reduction interventions reduce postoperative cardiovascular complications.

## 9. Conclusions

This review synthesizes evidence across three domains: stress pathophysiology, perioperative cardiovascular injury mechanisms, and clinical outcome data. Mechanistic evidence is well established and researched, stating that chronic stress is associated with HPA axis dysregulation, glucocorticoid resistance, sustained sympathetic activation, systemic inflammation, endothelial dysfunction, and hypercoagulability. Each of these mechanisms directly affects the pathways responsible for perioperative myocardial injury, arrhythmogenesis, and venous thromboembolism. General population epidemiology is equally strong, with large meta-analyses confirming that chronic psychological stress, depression, and occupational strain independently increase cardiovascular disease risk. Emerging perioperative observational data from the VISION cohort and several prospective surgical cohorts demonstrate consistent associations between preoperative psychological distress and adverse postoperative outcomes.

The convergence of mechanistic, epidemiological, and emerging clinical data presented in this review provides a compelling rationale for clinicians to consider preoperative psychological and psychosocial status as a potential dimension of perioperative cardiovascular risk. However, the current evidence base primarily reflects mechanistic plausibility and observational associations rather than a directly demonstrated perioperative causal pathway. Whether preoperative chronic stress independently contributes to postoperative cardiovascular events remains to be established through prospective studies with cardiovascular-specific endpoints and randomized controlled trials of targeted interventions.

## Figures and Tables

**Table 1:** Classification of stress-related constructs and measurement approaches discussed in this review.

Concept	Definition	Instruments/Measures	Timeframe	Limitations in Perioperative Context
Acute preoperative anxiety	Situational distress related to impending surgery	APAIS, STAI-State	Hours to days	Reflects state, not trait or chronic exposure
Recent psychological symptoms	Anxiety, depression, or nonspecific distress	K-6, HADS, GAD-7, PHQ-9	2–30 days	Single-timepoint measurement; does not confirm chronicity
Cumulative physiological burden	Multisystem dysregulation from sustained stress-response activation	Allostatic load (10-biomarker composite)	Months to years	Influenced by age, comorbidity, socioeconomic factors
Autonomic dysfunction	Sympathovagal imbalance	HRV, HRR, resting heart rate	Variable	Influenced by medications, fitness, comorbidities; not specific to stress

Abbreviations: APAIS, Amsterdam Preoperative Anxiety and Information Scale; GAD-7, Generalized Anxiety Disorder 7-item scale; HADS, Hospital Anxiety and Depression Scale; HRV, heart rate variability; HRR, heart rate recovery; K-6, Kessler Psychological Distress Scale (6-item); PHQ-9, Patient Health Questionnaire (9-item); STAI, State-Trait Anxiety Inventory.

**Table 2:** Stress-related biomarkers: Measurement characteristics, limitations, and potential perioperative applications.

Biomarker/Index	What It Measures	Advantages	Limitations	Potential Perioperative Application
Serum/salivary cortisol	Single-timepoint HPA axis output	Widely available; low cost; rapid turnaround	Subject to diurnal variation, pulsatile secretion, and acute situational influences; poor reflection of chronic stress	Limited utility for chronic stress assessment; may reflect acute preoperative anxiety
Diurnal cortisol slope	Pattern of cortisol decline across the day	Captures HPA axis circadian regulation; flatter slope associated with MACE	Requires multiple timed samples (4–6 per day); logistically demanding	Could identify patients with HPA axis dysregulation preoperatively
Hair cortisol concentration (HCC)	Cumulative cortisol secretion	Retrospective assessment of chronic HPA axis activity; not affected by acute stress or diurnal variation; associated with CVD and metabolic syndrome	Requires specialized immunoassay not available in most hospital labs; influenced by hair color, washing frequency, chemical treatment, BMI, and oral contraceptive use	Most promising neuroendocrine biomarker for chronic stress; could complement self-report instruments in preoperative assessment
Heart rate variability (HRV)	Autonomic nervous system balance	Non-invasive; reduced HRV associated with perioperative myocardial injury, mortality, and complications	Affected by medications (beta-blockers, anticholinergics), age, and comorbidities; no standardized perioperative protocol	Emerging evidence for perioperative risk stratification; could identify autonomic dysfunction not captured by standard risk models
Resting heart rate	Surrogate of autonomic tone	Universally available; included in consensus AL definition; independently predicts mortality	Nonspecific; influenced by medications, fitness, pain, fever, volume status; limited discriminatory power alone	Already routinely collected; included in 5-item consensus allostatic load index; low incremental cost
C-reactive protein (CRP)	Systemic inflammation	Widely available; low cost; included in consensus AL definition; associated with cardiovascular risk	Nonspecific; elevated by infection, trauma, surgery, obesity; cannot distinguish stress-related from other sources of inflammation	Routinely available preoperatively; elevated baseline CRP may identify patients with chronic inflammatory burden
Allostatic load (AL) composite index	Cumulative multisystem physiological dysregulation across cardiovascular, metabolic, immune, and neuroendocrine domains	Captures multisystem dysregulation; can be derived from routinely collected labs and vitals; associated with postoperative complications in surgical oncology cohorts	Components overlap substantially with established cardiovascular risk factors (hypertension, diabetes, dyslipidemia, obesity); not specific to psychological stress; no standardized scoring method	Potentially scalable preoperative screening tool; could identify patients with high cumulative physiological burden not captured by single-organ risk factors

Abbreviations: AL, allostatic load; BMI, body mass index; CRP, C-reactive protein; CVD, cardiovascular disease; HPA, hypothalamic–pituitary–adrenal; HRV, heart rate variability; MACE, major adverse cardiovascular events.

## Data Availability

No new data were created or analyzed in this study. Data sharing is not applicable to this article.

## Abbreviations

The following abbreviations are used in this manuscript:

HPA	Hypothalamic–pituitary–adrenal
GR	Glucocorticoid receptor
GCR	Glucocorticoid resistance
NF-kB	Nuclear factor kappa-light-chain-enhancer of activated B cells
eNOS	Endothelial nitric oxide synthase
NO	Nitric oxide
MINS	Myocardial injury after noncardiac surgery
VTE	Venous thromboembolism
MACE	Major adverse cardiovascular events
HADS	Hospital Anxiety and Depression Scale
K-6	Kessler Psychological Distress Scale (6-item)
GAD-7	Generalized Anxiety Disorder scale (7-item)
PHQ-9	Patient Health Questionnaire (9-item)
STAI	State-Trait Anxiety Inventory
